# 4324 Phase 1 Sterile Product Formulation and Manufacturing at Academic Medical Centers: An Introduction for Translational Researchers

**DOI:** 10.1017/cts.2020.161

**Published:** 2020-07-29

**Authors:** Robert Bruce MacArthur, Kenneth Rockwell, Amber Johnson, Roger Vaughan, Barry S. Coller

**Affiliations:** 1 Rockefeller University; 2 University of Pennsylvania

## Abstract

OBJECTIVES/GOALS: To facilitate the development of innovative injection products by providing translational researchers with a regulatory and manufacturing road map for producing small batch sterile products for Phase 1 research use. To leverage recent AMC investments in facility improvements and pharmacy training in the areas of sterile product production, testing, and environmental controls, that can be used to support production of phase 1 clinical trial supplies METHODS/STUDY POPULATION: Searching and organizing relevant data and information from web portals and databases in the following: areas: FDA, EMA, USP regulations, regulatory science, pharmaceutical formulation and analytics, supply vendors, analytical testing laboratories, and product testing laboratories. Present the information using a user friendly format including flow charts and development timelines, taking the perspective of the translational investigator. RESULTS/ANTICIPATED RESULTS:

- Choosing AMC resources vs outside consultants and vendors, leveraging local resources where possible
- Qualifying and monitoring suppliers, testing laboratories, in-house departments, and Contract Drug Manufacturing Organizations (CDMO)
- Bringing together the deliverables for the IND CMC section
- Where and how to leverage available products and science to simplify safe and reliable production

DISCUSSION/SIGNIFICANCE OF IMPACT: Use and utility of injectable drug products, both small molecule and biologics, is growing rapidly, and is projected to continue to escalate well into the next decade. This is due not only to advances in medicine, but also to improvements in AMC-based sterile product production, and a better understanding of small batch manufacturing methods. All three trends align in academic medical centers (AMC) and can be utilized by translational researchers, if they can understand the potential and regulatory requirements.

